# Low FT3/FT4 Ratio Is Linked to Poor Prognosis of Acute Myocardial Infarction in Euthyroid Patients with Type 2 Diabetes Mellitus

**DOI:** 10.3390/jcm11216530

**Published:** 2022-11-03

**Authors:** Xi He, Ruonan Gao, Yubin Wu, Kejun Wu, Jianmin Sun, Xintao Zhang, Libin Liu, Lianglong Chen

**Affiliations:** 1 Department of Cardiology, Fujian Institute of Coronary Artery Disease, Fujian Medical University Union Hospital, Fuzhou 350001, China; 2 Department of Endocrinology, Fujian Medical University Union Hospital, Fuzhou 350001, China

**Keywords:** FT3/FT4, type 2 diabetes mellitus, acute myocardial infarction, major adverse cardiac event, clinical outcome

## Abstract

This is an observational, retrospective, single-center study aimed to determine whether the free triiodothyronine (FT3) to free thyroxine (FT4) ratio was related to acute myocardial infarction (AMI) prognosis in individuals with type 2 diabetes mellitus (T2DM). A total of 294 euthyroid T2DM patients with new-onset AMI were enrolled. FT3/FT4 ratio tertiles were used to categorize patients into Group 1 (FT3/FT4 ≥ 4.3), Group 2 (3.5 ≤ FT3/FT4 < 4.3), and Group 3 (FT3/FT4 < 3.5). Major adverse cardiac events (MACE), including nonfatal myocardial infarction, target vessel revascularization (TVR), and cardiac mortality, served as the primary endpoint. Group 3 demonstrated a considerably higher incidence of MACE than the other two groups over the average follow-up duration of 21 ± 6.5 months (all *p* < 0.001). Multivariable Cox regression analysis showed that a low FT3/FT4 ratio was an independent risk factor for MACE after AMI (Group 1 as a reference; Group 2: hazard ratio [HR] 1.275, 95% confidence interval [CI]: 0.563–2.889, *p* = 0.561; Group 3: HR 2.456, 95% CI: 1.105–5.459, *p* = 0.027). Moreover, the area under the receiver-operating characteristic curve (AUC) indicates a good predictive value of FT3/FT4 ratio for MACE (AUC = 0.70). Therefore, in T2DM patients with AMI, a low FT3/FT4 ratio was strongly linked to poor prognosis.

## 1. Introduction

Type 2 diabetes mellitus (T2DM) is a common type of diabetes mellitus (DM), encompassing over 90% of all DM cases [1]. The rapid growth of the T2DM population has resulted in an increase in the burden on public health systems globally. According to the International Diabetes Federation (IDF) reports, DM and its complications caused 5.0 million deaths in adults aged 20–79 years worldwide in 2015 [2]. Notably, among the multiple complications of T2DM, cardiovascular complications are the leading cause of morbidity and mortality [3].

Abnormal glucose metabolism increases the risk of acute myocardial infarction (AMI) and leads to higher morbidity, mortality, and recurrence rates of the disease [4]. It acts independently or synergistically with other factors to worsen myocardial infarction prognosis [5]. Compared to individuals without T2DM, patients with T2DM have a greater risk of death from AMI [6]. Although efforts to improve the outcomes of AMI have resulted in an overall reduction in the death rate, neither of these studies considered patients with T2DM [7,8]. Therefore, the early assessment of AMI prognosis in patients with T2DM is vital for providing timely guidance for clinical therapy. 

A close relationship between thyroid hormones (THs) and AMI has been revealed over the past few decades. As hormones regulate metabolism, THs have a profound effect on myocardial contraction and arteriole resistance [9]. Recent studies in euthyroid individuals have demonstrated the prognostic significance of the free triiodothyronine (FT3) to free thyroxine (FT4) ratio for long-term AMI outcomes, and decreased values of FT3/FT4 have been reported to indicate a poor AMI prognosis [10,11]. However, few studies have investigated this relationship in T2DM patients. 

Thyroid disorders and DM bidirectionally influence each other [12]. THs play an important role in the regulation of food intake and glucose metabolism, whereas abnormal glucose metabolism conversely affects TH levels [13]. Based on this, we speculated that the FT3/FT4 ratio could also be a prognostic marker for AMI under diabetic conditions. Therefore, this study was carried out to explore the association between the FT3/FT4 ratio and AMI outcome in patients with T2DM, to facilitate the early identification of high-risk patients. 

## 2. Materials and Methods

### 2.1. Study Design and Patients

This study was an observational, retrospective, single-center investigation. Patients with new-onset AMI and either a history of T2DM or de novo T2DM diagnosed during hospitalization at Fujian Medical University Union Hospital between 1 January 2017 and 31 December 2019 were recruited. The hospital is a public tertiary hospital in Fujian Province, China. The requirements for inclusion were as follows: (1) individuals diagnosed with T2DM according to the WHO guidelines (fasting serum glucose level ≥7.0 mmol/L, 2 h serum glucose ≥11.1 mmol/L following an oral glucose tolerance test, or current use of blood glucose-lowering medication) and (2) a diagnosis of AMI in accordance with the American College of Cardiology/American Heart Association (ACC/AHA) (which includes the presence of chest pain, the elevation of the ST segment or new left bundle branch block (LBBB), and rising levels of serum troponin I (TnI)). Coronary angiography was performed to establish a definite diagnosis of AMI in all patients.

The exclusion criteria were as follows: (1) type 1 DM or other types of DM; (2) a record of thyroid diseases, such as hypothyroidism or hyperthyroidism; (3) a record of thyroid surgery; (4) taking thyroid-related medications such as amiodarone, levothyroxine, methimazole or propylthiouracil, or glucocorticoid; (5) a history of abnormal thyroid-associated antibodies, including thyroid peroxidase antibody (TPOAb) and anti-thyroglobulin antibodies (TGAb), or hormones, such as FT3, FT4, and thyroid-stimulating hormone (TSH) by laboratory examination; (6) the presence of any predominant severe systemic diseases; and (7) incomplete data that would affect the statistical results.

### 2.2. Demographics Collection and Biochemical Investigation

Demographic data, including age, body mass index, sex, systolic and diastolic blood pressures, heart rate, and current/past smoking status, were collected by well-trained physicians. The DM duration, presence of chronic conditions, such as hypertension, medication use, and average hospitalization days and cost (before Medicare reimbursement), were also recorded. The Killip classification of each patient was used to evaluate the heart failure severity in AMI. The Global Registry of Acute Coronary Events (GRACE) risk score, which is an acknowledged prognostic tool for AMI [14], was also calculated.

Blood samples were taken from patients on admission to assess the ratio of neutrophils to leukomonocytes (N/L), as well as the levels of the hemoglobin (Hb), high-sensitivity C-reactive protein (hsCRP), TnI, creatine kinase-MB (CK-MB), N-terminal pro-B-Type natriuretic peptide (NT-proBNP), and serum creatinine levels. Following admission, 12 h fasting blood samples were collected in the morning to detect the levels of fasting blood glucose, total cholesterol (TC), triglycerides (TG), high-density lipoprotein cholesterol (HDL-C), low-density lipoprotein cholesterol (LDL-C), Apoa1, ApoB, and plasma albumin (ALB) (HITACHI 912 Analyzer, Roche Diagnostics, Germany). Glycosylated hemoglobin A1c (HbA1c) concentrations were assessed using a standard method (HLC®-723G8 Analyzer, TOSOH, Tokyo, Japan). 

A chemiluminescent immunoassay was employed to detect the levels of serum FT3, FT4, and TSH (Immulite 2000, Siemens, Berlin, Germany). Normal ranges for FT3, FT4, and TSH are 3.53–7.37 pmol/L, 0.79–2.11 nmol/dl, and 0.56–5.91 mIU/L, respectively. FT3/FT4 ratio tertiles were applied to categorize the patients into three groups [11].

### 2.3. Coronary Angiography

All eligible patients underwent coronary reperfusion therapy, which included percutaneous coronary intervention (PCI) and coronary angiography (CAG) (Angiography equipment Azurion 3M15, Philips, Amsterdam, The Netherlands). CAG was performed using the Judkins method by two or more experienced associate senior physicians or interventional cardiologists. Coronary artery disease (CAD) was identified by measuring the luminal diameter of a main epicardial coronary artery. If a narrowing of more than 50% was found, this was considered to indicate CAD. The two-vessel disease was defined as either two lesions in the right coronary artery (RCA) or/and the main branch of the left coronary artery (LCA), or the presence of left main trunk lesions [15]. A multivessel disease was indicated by the presence of more than two severe coronary artery stenoses.

### 2.4. Echocardiography

Echocardiographic assessments were carried out using a cardiac color Doppler ultrasound scanner (EPIQ7, Philips, Amsterdam, The Netherlands) on the day of admission. The left ventricular ejection fraction (LVEF) was detected to evaluate cardiac function, which was assessed in accordance with the most recent recommendations from the American Society of Echocardiography using the modified Simpson’s method.

### 2.5. Follow-Up and Endpoints

The following methods were used to gather follow-up clinical data: interviewing patients via the telephone, routine outpatient clinic examinations, and reviewing medical records during rehospitalization. All patients were followed up for two years: every three months during the first year and every six months during the second year. Major adverse cardiac events (MACE), including nonfatal myocardial infarction, target vessel revascularization (TVR) (defined as percutaneous or surgical revascularization of prior treated vessels), and cardiac mortality, served as the study’s primary endpoints. 

### 2.6. Statistical Analysis

The Kolmogorov–Smirnov test was used to examine whether numerical data were normally distributed. Continuous variables were shown as mean ± standard deviation for a normal distribution and median (25th–75th percentile) for a non-normal distribution. The analysis of variance or Kruskal–Wallis H test was employed to compare differences in continuous data. Categorical variables were expressed as counts and percentages, and Pearson’s chi-square or Fisher’s exact test was used to analyze the difference between these variables. The Kaplan–Meier method and the log-rank test were performed to evaluate MACE occurrence during follow-up. The association between FT3/FT4 levels and clinical outcomes was examined using univariable and multivariable Cox proportional regression models, which were adjusted for clinically relevant variables, including age, sex, hypertension, dyslipidemia, LVEF, GRACE risk score, and HbA1C level. The hazard ratios (HR) with 95% confidence intervals (CI) were also calculated. Areas under the curve (AUC), which were classified as negligible (0.55), small (0.56–0.63), moderate (0.64–0.70), or strong (0.71), were used to determine discrimination and were assessed using a receiver-operating characteristic (ROC) curve. SPSS 22.0 for Windows (SPSS Inc., Chicago, IL, USA) and MedCalc version 11.4 (MedCalc Inc., Ostend, Belgium) were used for all statistical analyses. Two-tailed *p*-values less than 0.05 were regarded as statistically significant.

## 3. Results

### 3.1. Patients

**Figure 1:**
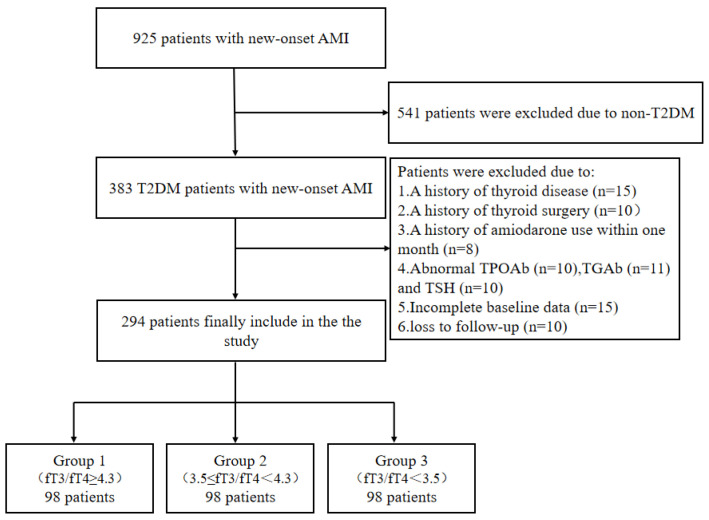
Study flowchart.

Of the 383 T2DM patients newly diagnosed with AMI, 89 patients were excluded for the following reasons: a history of thyroid disease (*n* = 15); a history of thyroid surgery (*n* = 10); amiodarone use within the last month (*n* = 8); a history of abnormal TPOAb (*n* = 10), TGAb (*n* = 11), and TSH (*n* = 10); incomplete baseline data (*n* = 15); and loss of follow-up (*n* = 10). Finally, 294 patients were enrolled (Figure 1). 

### 3.2. Baseline Characteristics

Table 1 displays the baseline characteristics of the enrolled patients. Based on the FT3 to FT4 ratio tertiles, patients were categorized into three groups: Group 1 (FT3/FT4 ≥ 4.3), Group 2 (3.5 ≤ FT3/FT4 < 4.3), and Group 3 (FT3/FT4 < 3.5). Group 3 had the highest proportion of patients with Killip class ≥ III when compared with Groups 1 and 2, and patients in Group 3 tended to have lower systolic blood pressures and levels of hemoglobin (Hb) and albumin (ALB). They also showed higher neutrophil/lymphocyte ratios (NLR) and higher levels of hsCRP and HbA1C. Moreover, a lower FT3/FT4 ratio was significantly associated with decreased LVEF values, increased NT-proBNP levels, and GRACE risk scores. Significant increases in hospital stays and expenses were also observed in Group 3.

### 3.3. Severity of Coronary Artery Disease and FT3/FT4 Ratio

The number of diseased vessels was determined using CAG to assess the severity of CAD in each group. Table 2 shows that CAD severity increased with decreasing serum FT3/FT4 ratio. Patients with lower FT3/FT4 ratios had a higher incidence of three-vessel disease and a lower incidence of single-vessel disease (*p* < 0.001). Moreover, the types of culprit vessels between groups did not differ significantly.

### 3.4. Clinical Outcomes

**Figure 2:**
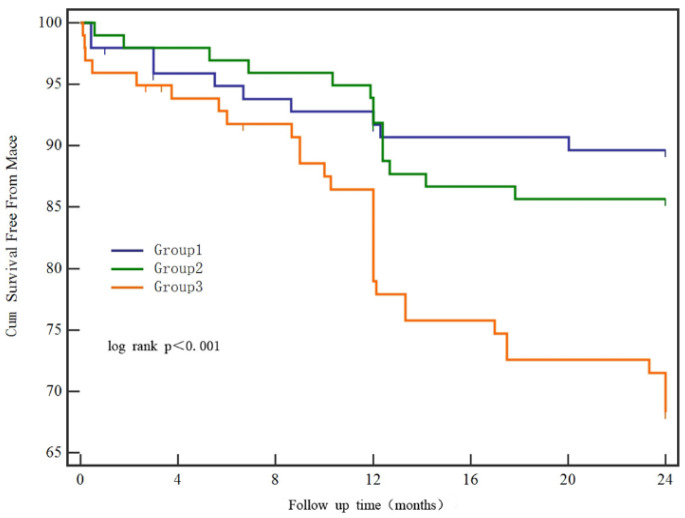
Kaplan–Meier survival analysis for MACE. Patients were divided according to decreasing tertiles of FT3/FT4 ratio (Group 1: FT3/FT4 ≥ 4.3, Group 2: 3.5 ≤ FT3/FT4 < 4.3, Group 3: FT3/FT4 < 3.5). FT3, free triiodothyronine; FT4, free thyroxine.

The average follow-up duration was 21 ± 6.5 months. During the follow-up, 10 patients were lost, and 54 MACEs occurred, including nonfatal myocardial infarctions in 25 patients, TVR in 17, and cardiac death in 12. As shown in Table 3, a low FT3/FT4 ratio in diabetic patients was significantly linked to a greater incidence of MACE (*p* < 0.001). The incidence of nonfatal myocardial infarction significantly increased in patients with a low FT3/FT4 ratio (*p* = 0.032). In addition, a low FT3/FT4 ratio tended to be correlated with an increased risk of TVR and cardiac death in patients with T2DM, although there was no statistical significance. Similar outcomes were found for MACE in the Kaplan–Meier survival analysis (log-rank *p* < 0.001) (Figure 2).

Univariable Cox hazard regression was performed to evaluate the potential risk of MACE. As shown in Table 4, factors that were significantly associated with a higher risk of MACE in the follow-up included LVEF level (HR: 0.966, 95% CI: 0.943–0.99, *p* = 0.005), the GRACE risk score (HR 1.011, 95% CI: 1.004–1.019, *p* = 0.004), HbA1C level (HR 1.154, 95% CI: 1.023–1.301, *p* = 0.02), and low FT3/FT4 ratio (HR 0.449, 95% CI: 0.313–0.646, *p* < 0.001). Multivariable analysis revealed that a low FT3/FT4 ratio was still linked to a greater risk of MACE after adjusting for covariates (Group 1 as reference; Group 2: HR 1.275, 95% CI: 0.563–2.889, *p* = 0.561; Group 3: HR 2.456, 95% CI: 1.105–5.459, *p* = 0.027) (Table 4).

### 3.5. Ability of FT3/FT4 Ratio and GRACE Risk Score to Predict MACE

**Figure 3:**
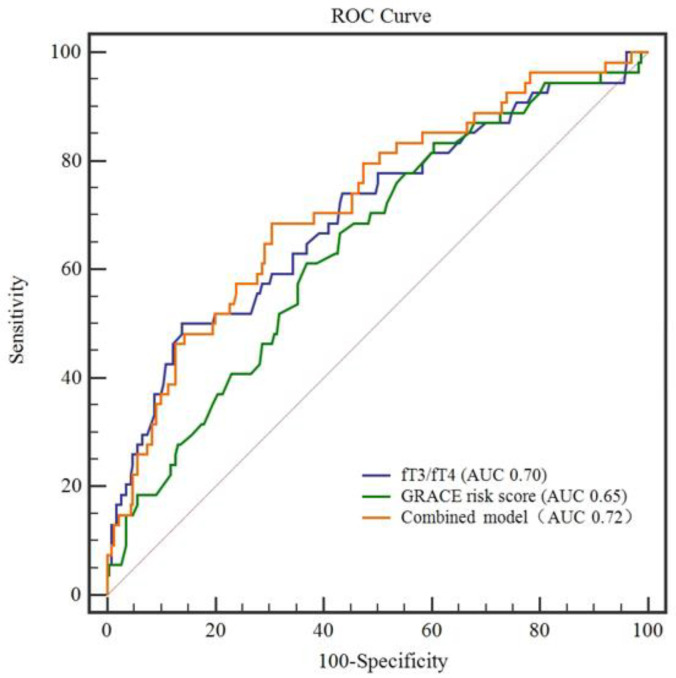
The receiver-operating characteristic (ROC) curve for FT3/FT4 ratio, the Global Registry of Acute Coronary Event (GRACE) risk score, and the combined value for predicting major adverse cardiac events (MACE). FT3, free triiodothyronine; FT4, free thyroxine.

A ROC curve was constructed to evaluate the predictive value of a low FT3/FT4 ratio for MACE, compared with that of the GRACE risk score, a known predictor of MACE, during a 2-year follow-up after AMI in patients with T2DM (Figure 3). The AUC of the FT3/FT4 ratio was 0.70 (95% CI: 0.644–0.754, *p* = 0.0427), and the ideal threshold was 3.13, with 50.0% sensitivity and 86.1% specificity. The AUC of the GRACE risk score for MACE prediction was 0.65 (95% CI: 0.587–0.702, *p* = 0.0413), and the optimal cutoff value was 141.5, with 61.1% sensitivity and 63.0% specificity under the cutoff value. Combining the FT3/FT4 ratio with the GRACE risk score yielded more accurate predictions for MACE than the GRACE risk score alone (AUC = 0.72, 95% CI: 0.665–0.772, *p* = 0.0397). However, compared to the FT3/FT4 ratio (AUC = 0.70), the combination with the GRACE risk score did not yield a significantly better prediction for MACE (*p* = 0.23). Combining the FT3/FT4 ratio and GRACE risk score had a sensitivity and specificity of 68.5% and 69.6%, respectively.

## 4. Discussion

CAD constitutes the main cause of death in patients with T2DM. Identifying patients with a high risk of MACE following AMI is vital and helps to make accurate clinical decisions early. In this study, a low FT3/FT4 ratio was linked to more severe myocardial dysfunction, which was assessed based on LVEF value, NT-proBNP level, Killip class, GRACE score, and hospital stay and cost. It was also correlated with a higher incidence of MACE during the 2-year follow-up after AMI, indicating the value of a low FT3/FT4 ratio in predicting the long-term prognosis of AMI in patients with T2DM.

There is a strong correlation between TH levels and the cardiovascular system. It is well known that THs can promote the beta-adrenergic positive chronotropic effect to increase the cardiac preload and ventricular filling pressure [16] and can regulate cardiac contractility, electrophysiological functions, and cardiac structure [9]. Previous studies have indicated that a low serum FT3/FT4 ratio might predict a poor prognosis for acute and chronic cardiovascular diseases [17,18]. FT3, one of the most biologically active THs, is converted from FT4 in the peripheral tissues, and a decreased FT3/FT4 ratio was observed after AMI in both humans and animals. Recent studies have reported that a decreased FT3/FT4 ratio was correlated with a higher incidence of cardiac-related and all-cause mortalities after AMI [11,19], indicating the importance of the FT3/FT4 ratio in predicting AMI outcome for patients with euthyroid. 

However, in patients with T2DM, the predictive value of the FT3/FT4 ratio for AMI outcome has not been fully evaluated. DM has been reported to affect THs through several different pathways. High levels of blood glucose can blunt the nocturnal TSH peak and impair the feedback of TSH to thyrotropin-releasing hormones [20]. A decreased uptake of T4 in peripheral tissues is also observed in diabetes and is associated with oxidative stress and excessive production of pro-inflammatory cytokines induced by diabetes [21]. Moreover, hyperglycemia can reduce deiodinase activity, impairing the conversion of T3 to T4 in peripheral tissues [22]. Clinical studies have indicated that FT3 levels are negatively associated with HbA1C levels in T2DM patients [23]. Furthermore, a meta-analysis based on 36 cross-sectional and case–control studies revealed that T2DM increases the incidence of subclinical hypothyroidism [24]. A recent study also reported significantly lower values of both FT3 and FT4 within the normal reference range in patients with T2DM, which could be elevated with the improvement of glucose control [25]. Since abnormal glucose metabolism leads to hypothalamic–pituitary–thyroid axis dysfunction, the significance of the FT3/FT4 ratio for AMI outcomes in T2DM patients remains elusive. 

Our results suggested that the FT3/FT4 ratio might indicate the severity and long-term outcome of AMI in T2DM patients. We found that it was negatively correlated with NT-proBNP levels and positively associated with LVEF values, which indicates the severity of AMI in patients with T2DM on admission. Group 3, which was characterized by a low FT3/FT4 ratio, had a greater rate of patients with a Killip class ≥ III. Similarly, the study by Su et al. found that patients with low FT3 levels had significantly higher NT-proBNP levels and incidence of in-hospital cardiovascular death [26]. A low FT3 level as an indicator of AMI severity has been reported in other studies [27,28]. Moreover, we showed that those with decreased FT3/FT4 ratios had greater levels of NLR and serum hsCRP and significantly lower levels of Hb and ALB, indicating a more severe degree of inflammation and worse clinical status. It is worth mentioning that we did not find any association between the FT3/FT4 ratio and TNI and CK-MB levels at baseline. The reasons for this inconsistency may be the limited sample size and different follow-up durations.

In our study, a low FT3/FT4 ratio significantly increased the incidence of MACE in AMI patients with T2DM during the 2-year follow-up period. This association remained significant even after adjusting for the influence of HbA1C by multivariable Cox regression analysis, indicating the significance of FT3/FT4 ratio for the prognosis of AMI in T2DM. It has been suggested that FT3 or FT4 alone cannot represent the overall changes of THs as accurately as the value of the FT3/FT4 ratio, and FT3/FT4 might be a more promising indicator to predict the outcomes in patients with DM under some clinical conditions [17,29]. In addition to the FT3/FT4 ratio, LVEF was also shown to be an independent predictor for MACE in the univariable regression analysis. A recent study showed that a severely reduced LVEF led to higher incidences of mortality and MACE of AMI in patients undergoing PCI [30], demonstrating the effectiveness of LVEF for evaluating the long-term outcome of AMI. However, multivariable regression analysis did not indicate a strong association between LVEF and MACE in the present study, which might be due to the limited sample size and follow-up duration. Moreover, the predictive value of HbA1C for MACE was not found to be significant during the follow-up after adjustment for multiple clinically relevant variables. These results were consistent with other studies [31,32], indicating that glucose variations or the mean amplitude of glycemic excursions had a better prognostic value for MACE than HbA1C.

The GRACE risk score is a reliable method of stratifying the severity of acute coronary syndrome [33] and is recommended by the current guidelines [34]. However, it seems to be limited in practice, as it does not wholly capture the indices (oxidative stress and inflammation, for example). Therefore, it does not fully represent the disease status in certain physiological and pathological conditions [35]. Univariable Cox regression analysis suggested that the GRACE risk score was strongly linked to the incidence of MACE in the present study. However, the predictive ability of the GRACE risk score was not significant after an adjustment in the multivariable Cox regression analysis. We further compared the prognostic values of the FT3/FT4 ratio combined with the GRACE risk score for the clinical outcomes of AMI in patients with T2DM. The ROC curve indicated that the FT3/FT4 ratio outperformed the GRACE score in predicting MACE during the 2-year follow-up. The combination of the FT3/FT4 ratio and GRACE risk score was better at predicting MACE in patients with T2DM than the GRACE risk score alone. These findings were in accordance with a previous study that indicated that the predictive power of the GRACE risk score could be enhanced by combining it with other indicators [35].

Some limitations exist in the current investigation. First, this was a single-center observational study, and the number of patients was relatively small, which might have limited the extrapolation of the study. Second, we did not know whether the FT3/FT4 ratio changed during follow-up because we lacked dynamic monitoring data of thyroid function. Third, the follow-up duration was relatively limited, and additional studies with longer follow-up periods are necessary to confirm our conclusion. In addition, ten patients were lost to follow-up, which might have caused a bias in the data. Despite these limitations, the present study may have clinical implications and provide significant guidance.

## 5. Conclusions

The present study suggests that a low FT3/FT4 ratio is strongly associated with the increased risk of MACE after AMI in T2DM populations. Combining the FT3/FT4 ratio with the GRACE risk score yielded more accurate predictions for MACE than the GRACE risk score alone. Routinely assessing the FT3/FT4 ratio on admission might help identify patients at high risk of AMI.

## Figures and Tables

**Table 1:** Demographic variables and baseline clinical characteristics.

	Group 1 (*n* = 98)	Group 2 (*n* = 98)	Group 3 (*n* = 98)	*p*-Value
Age, years	62.82 ± 11.87	66.34 ± 9.61	69.24 ± 10.64	<0.001
Male, *n* (%)	78 (79.6%)	72 (73.5%)	65 (66.3%)	0.111
BMI, kg/m^2^	24.86 ± 2.75	24.29 ± 2.76	24.1 ± 2.74	0.134
SBP, mmHg	131.77 ± 19.94	134.33 ± 21.22	125.18 ± 24.32	0.011 ^Δ^
DBP, mmHg	78.91 ± 12.33	80.01 ± 12.67	76.51 ± 15.16	0.178
Hypertension	61 (62.2%)	78 (79.6%)	76 (77.6%)	0.011
Previous MI	3 (3.1%)	6 (6.1%)	5 (5.1%)	0.59
Current/past smoking	62 (63.3%)	47 (48%)	46 (46.9%)	0.037
Myocardial infarction type				
STEMI, *n* (%)	46 (46.9%)	44 (44.9%)	41 (41.8%)	0.77
NSTEMI, *n* (%)	52 (53.1%)	54 (55.1%)	57 (58.2%)	0.77
Killip class ≥ III, *n* (%)	2 (2%)	3 (3.1%)	13 (13.3%)	<0.001 ^Δ^
Laboratory data				
hsCRP, mg/L	5.5 (2.13, 12.85)	6.66 (2.92, 19.9)	16.3 (5.71, 46.6)	<0.001 ^Δ^
NLR	3.0 (2.23, 4.89)	3.13 (2.27, 5.6)	4.63 (3.1, 7.38)	<0.001 ^Δ^
Hb, g/L	139.29 ± 16.77	132.27 ± 20.16	124.86 ± 18.44	<0.001 ^Δ^
TC, mmol/L	4.45 ± 1.08	4.25 ± 1.25	4.24 ± 1.1	0.37
HDL-C, mmol/L	0.95 ± 0.28	0.91 ± 0.22	0.95 ± 0.26	0.51
LDL-C, mmol/L	2.85 ± 0.95	2.79 ± 1.08	2.77 ± 0.95	0.33
apoA1, g/L	1.16 ± 0.22	1.1 ± 0.22	1.05 ± 0.24	0.06
apoB, g/L	1.02 ± 0.36	0.95 ± 0.38	0.96 ± 0.28	0.2
ALB, g/L	39.02 ± 4.46	37.47 ± 4.72	35.06 ± 4.21	<0.001 ^Δ^
Glucose, mmol/L	7.8 (6.59, 10.86)	8.8 (6.61, 11.63)	7.9 (6.28, 12.17)	0.554
HbA1C%	7.8 (6.8, 9)	7.65 (7, 8.8)	8.55 (7.2, 10.1)	0.021 ^Δ^
Cr, umol/L	73 (61, 90)	78 (64, 101)	87 (72, 104.5)	0.37
TNI, ng/mL	0.83 (0.23, 6.49)	1.2 (0.27, 10)	1.03 (0.33, 7.48)	0.474
CKMB, IU/L	23.4 (14.7, 97.7)	25.3 (16.8, 97.28)	26.5 (16.2, 53.78)	0.748
NT-proBNP, pg/mL	42 (180.75, 1106)	777.5 (218, 2243)	2217 (747, 5268)	<0.001 ^Δ^
LVEF, %	55.09 ± 10.82	52.92 ± 10.55	49.7 ± 11.55	0.003 ^Δ^
Medications, *n* (%)				
DAPT	96 (98%)	97 (99%)	93 (94.9%)	0.188
Statin	96 (98%)	97 (99%)	93 (94.9%)	0.188
ACEI/ARB	66 (67.3%)	75 (76.5%)	56 (57.1%)	0.015
β-blocker	68 (69.4%)	80 (81.6%)	76 (77.6%)	0.122
Insulin	21 (21.4%)	22 (22.4%)	30 (30.6%)	0.243
α-glucosidase inhibitor	61 (62.2%)	55 (56.1%)	40 (40.8%)	0.008
Insulin secretagogues	46 (46.9%)	40 (40.8%)	36 (36.7%)	0.345
Metformin	30 (30.6%)	17 (17.3%)	16 (16.3%)	0.025
DPP-4 inhibitor	2 (2)	5 (5.1%)	7 (7.1%)	0.241
Insulin sensitizer	29 (29.6%)	18 (18.4%)	16 (16.3%)	0.051
Duration of DM, months	1.6 (0.19, 4)	2 (0.43, 4)	4 (0.8, 6)	0.075
Grace risk score	122.08 ± 33.11	130.13 ± 29.17	155.14 ± 34.02	<0.001 ^Δ^
Hospital stay, days	8.5 (6.7, 12)	9 (7, 12)	11 (8, 15)	0.004 ^Δ^
Hospitalization costs, CNY	46,336 (38,196, 67,025)	46,048 (37,559, 72,722)	54,221 (44,210, 79,362)	0.016 ^Δ^

Patients were divided according to decreasing tertiles of FT3/FT4 ratio (Group 1: FT3/FT4 ≥ 4.3, Group 2: 3.5 ≤ FT3/FT4 < 4.3, Group 3: FT3/FT4 < 3.5); BMI, body mass index; SBP, systolic blood pressure; DBP, diastolic blood pressure; MI, myocardial infarction; STEMI, ST-segment elevation myocardial infarction; NSTEMI, non-ST-segment elevation myocardial infarction; hsCRP, high-sensitivity C-reactive protein; NLR, neutrophils/lymphocytes ratio; Hb, hemoglobin; TC, total cholesterol; HDL-C, high-density lipoprotein cholesterol; LDL-C, low-density lipoprotein cholesterol; ALB, albumin; HbA1c, glycated hemoglobin A1c; Cr, creatinine; TNI, troponin I; CK-MB, creatine kinase-MB; NT-proBNP, N-terminal pro-B-Type natriuretic peptide; LVEF, left ventricular ejection fraction; DAPT, dual antiplatelet therapy; ACEI, angiotensin-converting enzyme inhibitors; ARB, angiotensin receptor blockers; DPP-4, dipeptidyl peptidase-4; DM, diabetes mellitus; CNY, China Yuan. ^Δ^ *p* < 0.05, significant difference between Group 3 and the other two groups.

**Table 2:** Comparison of angiographic data of patients with acute myocardial infarction with different FT3/FT4 levels.

	Group 1 (*n* = 98)	Group 2 (*n* = 98)	Group 3 (*n* = 98)	*p*-Value
**Extent of CAD**				<0.001
Single-vessel disease	33 (33.7%)	30 (30.6%)	10 (10.2%)	
2-vessel disease	41 (41.8%)	42 (42.9%)	32 (32.7%)	
3-vessel disease	24 (24.5%)	26 (26.5%)	50 (57.1%)	
**Target vessel**				0.39
Left anterior descending artery	50 (51%)	43 (43.9%)	43 (43.9%)	
Left circumflex artery	18 (18.4)	23 (23.5%)	15 (15.3%)	
Right coronary artery	30 (30.6%)	32 (32.6%)	40 (40.8%)	
Left main artery	4 (4.1%)	8 (8.2%)	4 (4.1%)	

Patients were divided according to decreasing tertiles of the FT3/FT4 ratio (Group 1: FT3/FT4 ≥ 4.3, Group 2: 3.5 ≤ FT3/FT4 < 4.3, Group 3: FT3/FT4 < 3.5); CAD, coronary artery disease; FT3, free triiodothyronine; FT4, free thyroxine.

**Table 3:** Clinical outcomes in AMI patients based on fT3/fT4 ratio tertiles.

Event	Group 1 (*n* = 98)	Group 2 (*n* = 98)	Group 3 (*n* = 98)	*p*-Value
MACE	10 (10.2%)	14 (14.3%)	30 (30.6%)	<0.001 ^Δ^
Nonfatal MI	4 (4.1%)	7 (7.1%)	14 (14.3%)	0.032 ^Δ^
TVR	4 (4.1%)	4 (4.1%)	9 (9.2%)	0.21
cardiac death	2 (2%)	3 (3.1%)	7 (7.1%)	0.161

MACE, Major adverse cardiac events; AMI, acute myocardial infarction; MI, myocardial infarction; TVR, target vessel revascularization; Group 1: FT3/FT4 ≥ 4.3, Group 2: 3.5 ≤ FT3/FT4 < 4.3, Group 3: FT3/FT4 < 3.5; ^Δ^ *p* < 0.05, significant difference between Group 3 and the other two groups.

**Table 4:** Cox regression analysis of factors associated with major adverse cardiac events.

		Univariable Analysis			Multivariable Analysis		
		HR	95%CI	*p* Value	HR	95%CI	*p* Value
Age		1.012	0.987–1.038	0.343			
Male		0.829	0.444–1.548	0.556			
History of smoking		1.328	0.778–2.267	0.299			
Hypertension		0.812	0.435–1.515	0.512			
LVEF		0.966	0.943–0.99	0.005	0.978	0.953–1.004	0.102
Creatine		1.002	0.997–1.004	0.923			
Total cholesterol		0.914	0.719–1.162	0.463			
LDL-C		0.828	0.621–1.102	0.196			
Grace risk sore		1.011	1.004–1.019	0.004	1.003	0.994–1.011	0.561
HbA1C%		1.154	1.023–1.301	0.02	1.066	0.944–1.202	0.302
FT3/FT4		0.449	0.313–0.646	<0.001	0.526	0.347–0.795	0.002
	Group 1	1 (reference)	1 (reference)	...	1 (reference)	1 (reference)	...
	Group 2	1.374	0.61–3.094	0.443	1.275	0.563–2.889	0.561
	Group 3	3.315	1.62–6.784	0.01	2.456	1.105–5.459	0.027

LVEF, left ventricular ejection fraction; Cr, creatinine; LDL-C, low-density lipoprotein cholesterol; FT3, free triiodothyronine; FT4, free thyroxine; HR, hazard ratio; CI, confidence interval.

## Data Availability

Data are available on request from the corresponding authors.
